# Trend analysis of cardiovascular disease mortality, incidence, and mortality-to-incidence ratio: results from global burden of disease study 2017

**DOI:** 10.1186/s12889-021-10429-0

**Published:** 2021-02-25

**Authors:** Maedeh Amini, Farid Zayeri, Masoud Salehi

**Affiliations:** 1 Department of Biostatistics, School of Allied Medical Sciences, Shahid Beheshti University of Medical Sciences, Tehran, Iran; 2 Proteomics Research Center and Department of Biostatistics, School of Allied Medical Sciences, Shahid Beheshti University of Medical Sciences, Tehran, Iran; 3 Department of Biostatistics, Health Management and Economics Research Center, School of Public Health, Iran University of Medical Sciences, Tehran, Iran

**Keywords:** Cardiovascular disease, Mortality, Incidence, Mortality-to-incidence ratio, Generalized estimating equation methodology, Marginal model, Human development index, Trend

## Abstract

**Background:**

Cardiovascular diseases (CVDs) are one of the global leading causes of concern due to the rising prevalence and consequence of mortality and disability with a heavy economic burden. The objective of the current study was to analyze the trend in CVD incidence, mortality, and mortality-to-incidence ratio (MIR) across the world over 28 years.

**Methods:**

The age-standardized CVD mortality and incidence rates were retrieved from the Global Burden of Disease (GBD) Study 2017 for both genders and different world super regions with available data every year during the period 1990–2017. Additionally, the Human Development Index was sourced from the United Nations Development Programme (UNDP) database for all countries at the same time interval. The marginal modeling approach was implemented to evaluate the mean trend of CVD incidence, mortality, and MIR for 195 countries and separately for developing and developed countries and also clarify the relationship between the indices and Human Development Index (HDI) from 1990 to 2017.

**Results:**

The obtained estimates identified that the global mean trend of CVD incidence had an ascending trend until 1996 followed by a descending trend after this year. Nearly all of the countries experienced a significant declining mortality trend from 1990 to 2017. Likewise, the global mean MIR rate had a significant trivial decrement trend with a gentle slope of 0.004 over the time interval. As such, the reduction in incidence and mortality rates for developed countries was significantly faster than developing counterparts in the period 1990–2017 (*p* < 0.05). Nevertheless, the developing nations had a more rather shallow decrease in MIR compared to developed ones.

**Conclusions:**

Generally, the findings of this study revealed that there was an overall downward trend in CVD incidence and mortality rates, while the survival rate of CVD patients was rather stable. These results send a satisfactory message that global effort for controlling the CVD burden was quite successful. Nonetheless, there is an urgent need for more efforts to improve the survival rate of patients and lower the burden of this disease in some areas with an increasing trend of either incidence or mortality.

## Background

Cardiovascular disease (CVD) is known as the leading cause of global death and one of the most serious health problems throughout the world. Commonly, CVD can refer to a class of diseases that involves the heart or blood vessels. This disease consists of stroke, heart failure, hypertensive heart disease, rheumatic heart disease, peripheral arterial disease, and a number of other vascular, and cardiac problems. However, CVD which has been recognized as the leading cause of morbidity and mortality, is an important contributor to the cost of medical care [1]. In 2016, CVD was responsible for nearly one-third of all deaths across the globe [2]. Over the last decades, although the age-standardized mortality rates of CVD declined by 27.3%, the number of deaths increased by 42.4% from 1990 to 2015 [3]. On the other hand, CVD led to over 17 million deaths, 330 million years of life lost and 35.6 million years lived with disability in 2017 worldwide [4, 5]. Meanwhile, it was projected that CVD would be the cause of more than 23 million deaths in 2030 around the world [6]. In the past decades, although developing countries have experienced higher rates of death from CVD, this disease was more incident in developed countries [7]. According to the World Health Organization (WHO), over three-quarters of CVD deaths have occurred in low- and middle-income countries which is a growing epidemic problem in recent years [8]. The high burden of CVD can be attributed to other related factors such as diabetes, obesity, lack of physical activity, hypertension, unhealthy diet, and excessive alcohol consumption [9]. Today, CVD is responsible for a remarkable reduction in quality of life and life expectancy and also imposes huge costs on health systems in different countries.

Accurate estimation of disease burden plays a key role in establishing convenient public health policies. To measure the burden of CVD, different outcome parameters can be used. There are some indices such as prevalence, incidence, mortality, and survival which can provide valuable information about the current situation and help the policy makers to organize the available resources. In general, the incidence and mortality rates of CVD vary from region to region because of several factors like lifestyle, dietary habits, appropriate health care accessibility, and so on. For example, people with a lower level of education in low-income and middle-income countries have a higher incidence of mortality from CVD [10]. Notably, assessing the patterns of differences in disease incidence and mortality rates is of international interest owing to highlighting the trends and regional differences which help to figure out the etiology of CVD. In this context, the Mortality-to-Incidence Ratio (MIR) is an alternative index that has been commonly used to evaluate the burden of disease by presenting mortality after accounting for incidence. Clearly, this ratio determines whether a country or a region has a higher or lower mortality for a specific condition, normalized to its incidence. Additionally, it is a simple and common proxy for the 5-year relative survival of the patients [11, 12]. More specifically, the MIR could be used as a helpful indicator for screening and treating some diseases.

During recent years, various studies have indicated that the incidence and mortality can be affected by economic and social disparities which reflect the regional disparities in human development. Human development index (HDI) is one of the important approved indicators for determining the progress, living conditions, and human development of different countries. This index, which is specified by the World Bank, combines a country’s per-capita gross national income (GNI) [13]. The HDI comprises socio-economic variables that affect health and national development factors of people such as political rights, mortality rate, and education. HDI is calculated as a simple arithmetic mean of three indexes reflecting longevity, knowledge, and standard of living. Longevity is measured by life expectancy at birth, knowledge is quantified by potential years of education, and standard of living is represented by income or gross national product (GDP) per capita [14].

Over the previous decades, several epidemiological studies have examined the incidence and mortality trends of CVD in different parts of the world. In a study conducted by Smolina et al., it was reported that a 50% decline in age standardized mortality occurred in England between 2002 and 2010 [15]. In another study, Sidney et al. showed that the CVD mortality rate decreased since 2011 in United States which might be due to remarkable improvement in population-level CVD prevention [16]. It was also revealed that the trend of age standardized CVD mortality rates in high-income regions has dramatically reduced during the last 30 years whereas this trend has slightly declined or even increased in most low and middle income countries [17] .

Up to now, there are no published studies about assessing the time trend patterns of CVD incidence and mortality rates in various parts of the world via more advanced statistical techniques. Besides, no published research is available about evaluating the temporal trend of CVD MIR globally to date. Also, No evidence based document is currently available about the association between the trend of CVD incidence, mortality, and MIR and HDI using more complex statistical modeling approaches. However, examining time trends of CVD indices across the world, as well as investigating the effect of development factor on the indices trends can be important to look into avenues for further improvement. Thus, the novelty of the present study lies in achieving the following specific objectives: a) to assess the mean trends in age-standardized CVD incidence, mortality, and MIR rates for the total world and different super regions of the Institute for Health Metrics and Evaluation (IHME) in the period 1990–2017 and b) to determine the longitudinal relationship between age-standardized CVD incidence, mortality, and MIR rates and HDI during the specified time interval using the marginal modeling approach which has a three-part specification in terms of a regression model for the mean response. To achieve these goals, the age-standardized CVD incidence and mortality rates data was extracted from the Global Burden of Disease (GBD) Study 2017.

## Methods

### Data sources

In this study, the information from the GBD free online database (GBD study 2017) on age-standardized CVD mortality and incidence rates per 100,000 persons was extracted from 1990 to 2017 for both genders. GBD is an international cooperative project that globally, regionally, and nationally estimates the disease burden for total countries and is managed by the IHME which provides the world’s most important health measurements. The GBD data sets are gathered and analyzed by a consortium of more than 1800 researchers in more than 100 countries. GBD is the most comprehensive worldwide epidemiological study which contains burden of disease indices such as incidence, mortality, prevalence, years of life lost (YLL), years lived with disability (YLD), and disability adjusted life years (DALY) for different diseases and injuries. Furthermore, the GBD data is provided by different organizations like WHO Global Health Observatory, World Bank Open Data, and the Inter-university Consortium for Political and Social Research [4, 5]. The IHME has classified all countries into seven distinct parts referred to super region according to geographical and economic criteria: Central Europe, Eastern Europe, and Central Asia (CEEECA) including 29 countries, High Income (HI) including 35 countries, Latin America and Caribbean (LAC) including 31 countries, North Africa and Middle East (NAME) including 21 countries, South Asia (SA) including 5 countries, Southeast Asia, East Asia, and Oceania (SAEAO) including 28 countries, and Sub-Saharan Africa (SSA) including 46 countries.

Human Development Index, introduced by the United Nations Development Programme (UNDP) in 1990, is defined as the average achievement of three dimensions including life expectancy at birth as the health indicator, gross national income per capita as the economic indicator, and mean and expected years of schooling for school-age children and average years of schooling in the adult population as the educational indicator. The geometric mean of the three indicators provides the aggregate value of HDI for a given country in a year [18]. The numerical value of the HDI is ranged from zero (worst) to one (best). This socio-economic index suggests the most up-to-date information on global development and contains national, regional, and global estimates. In this study, countries with HDI values of less than 0.788 were considered as developing and those with HDI values of 0.788 or higher as developed [14]. The HDI data for each country in the period of 1990–2017 was obtained from updates of the UNDP database [19].

### Variables under study

In the present research, the considered outcomes under study were the CVD incidence, mortality, and mortality-to-incidence ratio for each of the countries in the years 1990 to 2017. Calculation of the MIR provides an alternative means that could expand the interpretation and understanding of the relationship between the burden of disease by presenting mortality and incidence. This measurement can help us to determine the demographic, environmental, and social factors which might lead to changes in mortality rates according to incidence rates. Notably, the MIR can explore and address the hidden differences in disease incidence [20]. Further, using this index, one can forecast burdens, trends, and retrospectively evaluate populations with shorter or longer five-year survival. This information can be useful for disease prevention, diagnosis proficiency, treatment effectiveness, and making decisions to gain better survival [21, 22]. A retrospective study found that there is a positive correlation between the MIR values and healthcare system ranking for different countries [20].

In the current study, the MIR value for each country was calculated as the publicly available data of age-standardized mortality rate divided by age-standardized incidence rate of CVD in both genders from the GBD study 2017, separately in each year of the study. As mentioned, this index was used as a valid proxy for 5-year relative survival of CVD patients. In addition, for each country the development factor as a binary variable (0 = Developing, 1 = Developed) was considered as the independent predictor of incidence, mortality, and MIR in the statistical analysis process.

### Statistical analysis

Firstly, summary statistics related to CVD incidence, mortality, and MIR were provided for each IHME super region from 1990 to 2017. Likewise, the average annual trends of the CVD incidence, mortality, and MIR indices were exhibited graphically by super region and development factor over the study period.

In the next step, the marginal modeling approach and Generalized Estimating Equations (GEE) methodology utilized to assess the longitudinal effect of development factor on incidence, mortality, and MIR indices. The GEE is a population-level approach which enables us to obtain the population-averaged estimates of the model parameters [23]. To investigate the time trend of CVD burden indices, the following marginal model was fitted, separately for developed and developing countries:

1 $$ {\mu}_{ij}={\beta}_0+{\beta}_1\ {time}_{ij} $$

where *μ*_*ij*_ represents the mean incidence, mortality, or MIR for the *i*^th^ country (*i* = 1,2, …,195) in the *j*^th^ year of the study (*j* = 0,1, …,27). Here, *β*_0_ indicates the mean starting value of the index (intercept) and *β*_1_ is the model slope which shows the mean annual change in these indices. The time covariate shows the study time point (time = 0, 1, …,27 as a proxy for year = 1990, 1991, …, 2017). Notably, in some areas, the mean trend of the outcome under study might have a non-linear form. To capture the non-linear nature of these trends, the following spline model could be utilized as:

2 $$ {\mu}_{ij}={\beta}_0+{\beta}_1\ {time}_{ij}+{\beta}_2{\left({time}_{ij}-{t}^{\ast}\right)}_{+} $$

where *t*^∗^ indicates the turning point in the mean trend plot and the covariate (*time*_*ij*_ − *t*^∗^)_+_ = 0 if *time* ≤ t^*^ and (*time*_*ij*_ − *t*^∗^)_+_ = *time*_*ij*_ − *t*^∗^ if *time*_*ij*_ > *t*^∗^. It should be noted that the interpretation of the estimates in marginal models is rather analogous to the common simple linear regression models. All analyses were conducted using the STATA 14.0 (https://www.stata.com) and SPSS 22.0 (SPSS Inc., Chicago, IL, USA) softwares. *P*-values less than 0.05 were regarded statistically significant.

## Results

**Fig. 1:**
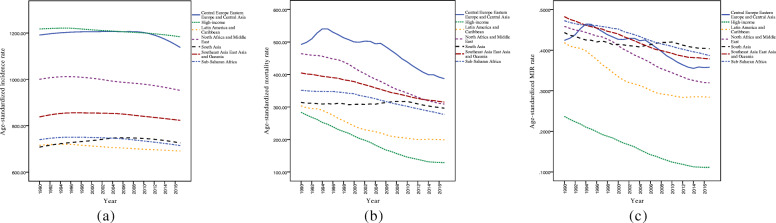
Mean trends of CVD (**a**) incidence, (**b**) mortality, and (**c**) MIR rates per 100,000 individuals by IHME super regions in the period 1990–2017

A total of 195 countries were included in the current study to assess the trend of CVD incidence, mortality, and MIR in the period 1990–2017. Figure 1 represents these trends in different IHME super regions. As can be seen in Fig. 1, the incidence rates were rather steady for HI, LAC, NAME, SSA, and SAEAO over the 28-year study period. Also, the countries in CEEECA had a moderate rise in CVD incidence over time until 2010, but then they had reached a steep decrementing trend during 2010–2017. Likewise, SA experienced a marked increasing trend of CVD incidence between 1990 and 2006, but it had a stable trend from 2006 to 2017. On the other hand, with the exception of SA region which had an increasing trend between 2006 and 2010, HI, LAC, NAME, SAEAO, and SSA regions experienced a downward trend in mortality rates. Additionally, the CEEECA countries had an upward trend in their CVD death between 1990 and 1995 and a declining pattern during the years 1995–2017. Accordingly, trends in age-standardized MIR from CVD among SSA, SAEAO, NAME, LAC, and HI countries declined sharply throughout the period. In CEEECA, the pattern of MIR was incremental in 1990–1994 with a peak in 1994 and a subsequent reduction from 1994 to 2017. Furthermore, the trend of MIR from CVD between 2004 and 2010 was, in fact, increasing and started to decrease consistently after these years in SA.

Table 1 shows the descriptive statistics for CVD incidence, mortality, and MIR in each IHME super region and total countries in 5-year intervals (and year 2017). Based on the Table, one can observe that countries in the HI region have experienced the highest incidence rates and the lowest mortality rates and MIRs in the period 1990–2017. In this time interval, SA countries had the lowest incidence rate at the starting point of the study and after the year 1995, the lowest incidence rate was related to LAC. From the mortality rate point of view, the CEEECA super region had the highest values in the study period. In addition, the maximum MIR value belonged to the SAEAO super region in the year 1990, while the highest ratio was related to SA in the ending year of the study. Besides, at the beginning and ending of the study, the minimum MIR value of CVD was observed among HI countries. Moreover, last row of Table 1 displays that all countries had a relatively rapid increment in mean IR until 1997 and then continued with a sharp reduction until the ending year of the study.

**Table 1:** Mean trend of CVD incidence, mortality, and MIR per 100,000 by IHME super region over 1990–2017

Super region^a^	index	Year						
		1990	1995	2000	2005	2010	2015	2017
CEEECA	Incidence	1191.2 (198.1)^b^	1202.4 (182.7)	1205.6 (171.4)	1206 (175.3)	1202.1 (181.5)	1162.9 (187.6)	1137.5 (191.8)
	Mortality	492.2 (66.9)	540.3 (107.9)	499.9 (115.1)	495.5 (144.2)	438.6 (129.2)	398.8 (123.5)	387.1 (120.8)
	MIR	0.42 (0.09)	0.46 (0.12)	0.42 (0.12)	0.42 (0.14)	0.37 (0.13)	0.35 (0.13)	0.35 (0.14)
HI	Incidence	1217.3 (168.5)	1221.1 (179.9)	1213.6 (198.4)	1206.8 (208.7)	1200.4 (209.3)	1189.5 (203.6)	1183.7 (199.6)
	Mortality	283.7 (52.4)	246.8 (44.8)	210.5 (42.2)	175.1 (37.9)	145.8 (33.5)	130.2 (31.8)	128.9 (32.3)
	MIR	0.23 (0.05)	0.20 (0.04)	0.17 (0.04)	0.14 (0.03)	0.12 (0.03)	0.11 (0.03)	0.11 (0.03)
LAC	Incidence	715.8 (57.8)	720.03 (57.7)	712.3 (55.02)	705.2 (54.2)	698.6 (51.2)	693.2 (48.9)	691.2 (48.2)
	Mortality	303.3 (94.2)	282.3 (84.1)	241.5 (77.7)	219.8 (73.1)	203.8 (71.5)	200.2 (74.1)	198.7 (71.8)
	MIR	0.41 (0.09)	0.38 (0.08)	0.33 (0.08)	0.30 (0.08)	0.28 (0.08)	0.28 (0.09)	0.28 (0.08)
NAME	Incidence	1000.3 (110.1)	1011.8 (115.7)	1005.1 (111.3)	989.5 (99.6)	979.1 (86.8)	961.4 (76.1)	952.3 (72.7)
	Mortality	464.1 (126.2)	451.9 (136.9)	420.5 (137.1)	378.3 (130.9)	342.8 (127.1)	315.1 (125.7)	308.9 (126.1)
	MIR	0.45 (0.08)	0.43 (0.09)	0.41 (0.09)	0.37 (0.09)	0.34 (0.10)	0.32 (0.10)	0.31 (0.11)
SA	Incidence	707.8 (71.4)	725.2 (85.4)	735.6 (98.1)	748 (102.1)	745.1 (98.4)	733.1 (87.4)	725.8 (81.2)
	Mortality	314 (38.2)	310.3 (70.7)	308.1 (81.4)	311.7 (89.4)	316.9 (85.5)	300.2 (77.3)	296.2 (77.1)
	MIR	0.44 (0.02)	0.42 (0.04)	0.41 (0.05)	0.41 (0.06)	0.42 (0.06)	0.40 (0.05)	0.40 (0.05)
SAEAO	Incidence	838.1 (78.03)	854.6 (79.1)	854.9 (82.1)	851.9 (87.3)	840.5 (86.1)	828.4 (87.9)	823.2 (90.1)
	Mortality	404.7 (118.7)	392.5 (119.9)	378.1 (123.7)	354.1 (127.6)	333.8 (131.5)	319.5 (128.8)	314.8 (126.2)
	MIR	0.48 (0.12)	0.45 (0.12)	0.43 (0.12)	0.41 (0.12)	0.39 (0.12)	0.38 (0.13)	0.37 (0.12)
SSA	Incidence	740.1 (43.7)	750.5 (45.9)	749.4 (46.1)	745.1 (46.2)	734.1 (43.3)	720.6 (38.2)	714.5 (35.6)
	Mortality	351.5 (84.7)	347.4 (81.6)	340.6 (68.1)	320.8 (68.2)	302.1 (69.2)	284.3 (65.4)	276.7 (62.2)
	MIR	0.47 (0.09)	0.46 (0.09)	0.45 (0.07)	0.42 (0.07)	0.40 (0.08)	0.39 (0.08)	0.38 (0.08)
Global	Incidence	928.7 (239.8)	938.3 (239.2)	935.6 (240.3)	930.5 (241.8)	922.3 (243.1)	906.5 (237.2)	898 (233.2)
	Mortality	371.9 (115.7)	365.1 (134.1)	339.1 (133.3)	316.1 (140.9)	288.7 (132.5)	269.8 (125.2)	264.3 (121.8)
	MIR	0.41 (0.12)	0.40 (0.13)	0.37 (0.13)	0.35 (0.13)	0.32 (0.13)	0.31 (0.13)	0.30 (0.13)

^a^ *CEEECA* Central Europe, Eastern Europe, and Central Asia, *HI* High Income, *LAC* Latin America and Caribbean, *NAME* North Africa and Middle East, *SA* South Asia, *SAEAO* Southeast Asia, East Asia, and Oceania, *SSA* Sub-Saharan Africa. ^b^ Mean (Standard deviation)

**Fig. 2:**
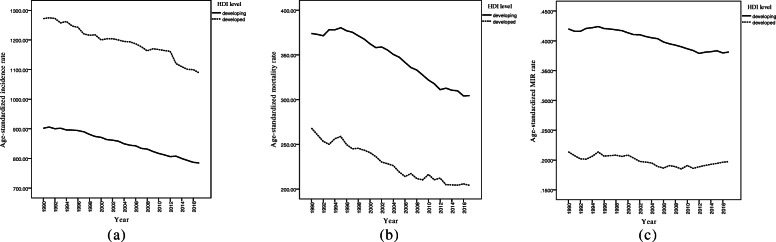
Mean trends of CVD (**a**) incidence, (**b**) mortality, and (**c**) MIR rates per 100,000 individuals by development factor in the period 1990–2017

**Fig. 3:**
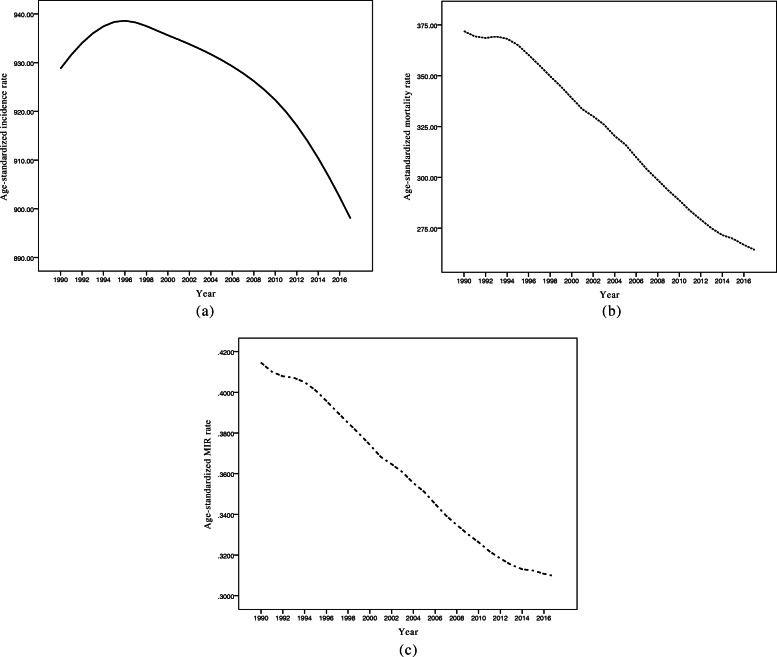
Mean Trends of CVD (**a**) incidence, (**b**) mortality, and (**c**) MIR rates per 100,000 individuals for total world countries in the period 1990–2017

The trends of incidence rate (IR), mortality rate (MR), and MIR, separately for developing and developed countries are depicted in Fig. 2. As seen, it seems that CVD IR and MR fell substantially and MIR slightly decreased in both developed and developing countries over the follow-up time. The descriptive statistics for CVD IR, MR, and MIR values, separately for the developing and developed countries in selected years are summarized in Table 2. With respect to Table 2, one can observe that developing countries had lower CVD IR and higher MR and MIR values compared to developed countries in the study period. Figure 2 illustrates that the trends in IR and MR decreased with a relatively rapid slope in both developed and developing areas. Nonetheless, the rates of MIR had rather fixed decreased trends with gentle slopes over and stayed steady till 2017 across the countries. Also, as can be seen, there were substantial gaps between trends of incidence, mortality, and MIR. In addition, Fig. 3 demonstrates the age-standardized IR, MR, and MIR trends for the entire world from 1990 to 2017. As shown in Fig. 3, the mean CVD MR and MIR trend declined steeply over the study interval.

**Table 2:** Mean trend of CVD incidence, mortality, and MIR per 100,000 by development factor over 1990–2017

Countries	Index	Year						
		1990	1995	2000	2005	2010	2015	2017
Developing	Incidence	901.8 (225.6)^a^	895.8 (214.8)	880.9 (203.4)	896.4 (210.1)	888.7 (217.8)	878.9 (217.3)	877.1 (230.7)
	Mortality	373.9 (116.6)	380.4 (136.3)	362.4 (129.8)	347.4 (137.1)	321.8 (127.3)	309.7 (120.1)	304.4 (117.8)
	MIR	0.42 (0.10)	0.42 (0.10)	0.41 (0.10)	0.40 (0.10)	0.38 (0.10)	0.38 (0.10)	0.38 (0.10)
Developed	Incidence	1272.1 (185.2)	1246.9 (192.7)	1200.1 (223.1)	1193.7 (219)	1167.6 (236.4)	1100.6 (250.1)	1088.8 (243.9)
	Mortality	268.0 (43)	258.7 (76.6)	240.7 (79.8)	219 (91.9)	216.2 (115.6)	204.2 (106.4)	204.1 (107.8)
	MIR	0.21 (0.04)	0.21 (0.08)	0.20 (0.08)	0.18 (0.08)	0.19 (0.10)	0.19 (0.10)	0.19 (0.10)

^a^ Mean (Standard deviation)

Apparently, as Fig. 2 indicates, since the mean trend of all the three indices seems to be linear for both the developed and developing countries, the described model [1] was applied to estimate the intercept and slope of the mean trends. Table 3 provides the obtained estimated parameters based on GEE approach. The estimates for the incidence rates tell us that developed countries had an intercept of about 378 per 100.000 higher than developing countries. This means that the mean IR in developed countries was about 378 per 100.000 higher than developing countries in 1990. In addition, the estimated slope for developed countries was about 1.8 times of the estimate for developing countries over time. In other words, the developed countries had steeper IR reduction compared to developing countries. Regarding the estimates for MRs, one can conclude that developed countries had an intercept of about 105 per 100.000 lower than developing countries. This means that the mean MR in developed countries was about 105 per 100.000 lower than developing countries in the starting year of the study. The nearly equal estimated slopes for mean MRs in developed and developing countries (− 2.87 vs. -2.69) indicate a relatively similar mean MR reduction for countries in both development conditions. Finally, the estimates for the mean MIRs illustrate that in developed countries, the mean MIR was about 0.20 lower than developing countries in 1990. Likewise, the estimated slopes for the mean MIR in developing countries were two times of the estimate in developed countries from 1990 to 2017 (− 0.002 vs. -0.001).

**Table 3:** Parameter estimates from modeling the mean trend of CVD incidence, mortality, and MIR by development factor between 1990 and 2017

HDI^a^ level	Index	Parameter	Estimate	SE^b^	*P* value
Developing	Incidence	Intercept	904.51	19.50	< 0.001
		Time	−3.71	0.60	< 0.001
	Mortality	Intercept	381.14	11.24	< 0.001
		Time	−2.69	0.41	< 0.001
	MIR	Intercept	0.42	0.01	< 0.001
		Time	−0.002	0.0005	< 0.001
Developed	Incidence	Intercept	1282.77	40.40	< 0.001
		Time	−6.66	1.44	< 0.001
	Mortality	Intercept	276.58	10.96	< 0.001
		Time	−2.87	0.54	< 0.001
	MIR	Intercept	0.22	0.01	< 0.001
		Time	−0.001	0.0005	0.009
Total World	Incidence	Intercept	903.19	16.74	< 0.001
		Time	4.31	0.70	< 0.001
		(Time-6)	−5.84	0.67	< 0.001
	Mortality	Intercept	370.10	8.35	< 0.001
		Time	−3.82	0.23	< 0.001
	MIR	Intercept	0.42	0.009	< 0.001
		Time	−0.004	0.0003	< 0.001

^a^*HDI* Human Development Index. ^b^*SE* Standard error

Also the mean trends of IR, MR, and MIR was modeled for total countries (Table 3). According to the mean trends in Fig. 3, since the mean IR trend has a non-linear shape, the illustrated model [2] in the Methods section was applied with *t*^∗^ = 6 (due to the presence of the turning point in the year 1996) for this index. In addition, owing to the observed linear mean trends for MR and MIR, the linear model [1] was utilized to estimate the intercepts and slopes of mean trends for these two indices. Based on these results, the estimated intercept for CVD incidence rate in all countries was about 903.19 per 100,000 people in 1990 followed by a positive slope of 4.31 per 100.000 until the year 1996 and thereafter a slope of 4.31*time*–5.84 (*time*–6) from 1996 to 2017. Clearly, the mean CVD incidence rate has increased annually by 4.31 per 100.000 until 1996 and then has diminished by a factor of about 1.53 per year of follow-up (4.31–5.84 = − 1.53). Furthermore, the fitted model revealed a statistically significant downward mean mortality rate with an annual reduction of 3.82 per 100,000 people between 1990 and 2017 for total countries. Likewise, the mean MIR has globally decreased with an annual reduction of 0.004 over the 28-year study period.

## Discussion

Cardiovascular diseases as a major concern for global health, have been the leading cause of global mortality since 1980. Most epidemiological studies conducted on incidence and mortality rates due to CVD are descriptive or cross-sectional and identifying the time trends of CVD incidence along with mortality are not well documented. Additionally, the effects of development factor and geographic location on CVD incidence, mortality, and MIR have not been discussed and approved in any studies. Therefore, to characterize and make comparisons this diversity in distinctive regions with regard to their trends from 1990 to 2017, using proper statistical technique seems to be necessary with a high degree of accuracy. This matter was investigated in this long-term study.

The results from the GBD 2017 data for CVD incidence indicate that all super regions had a rather steady decreasing trend during the interval 1990–2017, except CEEECA and SA super regions which experienced an increasing trend before the year 2010 followed by a downward trend until the ending year of the study. This study is fairly unique in this field (global trend analysis of CVD incidence), although a few related studies are available on the subject of the global incidence trends of CVD subtypes. For instance, in a study by Khan et al., it was found that the incidence of age-standardized ischemic heart disease has decreased globally in the period 1990–2017. They also stated that this reduction may be partly due to increasing global awareness about healthy lifestyle [24]. Based on another study by Avan et al., the age-standardized incidence rate of stroke has declined worldwide by 11.3% during the same time interval [25]. Nevertheless, reviewing the available literature on regional incidence trend of CVD subtypes in different parts of the world may lead to more controversial results. For example, Wu et al. demonstrated that the incidence rate of congenital heart disease (CHD) remained stable from 1990 to 2017 at the global level, whilst a decreasing trend was mainly found in Central or South America and Africa. According to their opinion, the decrement in incidence was largely owing to the rise in the termination rate following a prenatal diagnosis of CHD in these regions. By contrast, they reported an increment in the trend of CHD incidence in countries located in Western Europe over the last few decades. They concluded that an increase in ventricular septal defect and atrial septal defect subtypes might be the reason for this increment in the above mentioned countries [26]. In addition, as reported by an earlier analysis of GBD data, the incidence of stroke has declined in most regions particularly Southern Latin America from 1990 to 2016, whereas the incidence has increased in East Asia and Southern Sub-Saharan Africa [27]. In general, educating people about healthy lifestyle, better global accessibility to medication for controlling the CVD risk factors and improvement in CVD prevention strategies might be considered as the most important reasons for the downward global trend of CVD incidence.

Based on the findings regarding mortality trends, it is apparent that nearly all countries have experienced a significant declining trend over 1990 to 2017 with an annual reduction of 3.82 per 100,000 individuals. This is particularly prominent in HI countries, with the largest decrease in CVD mortality. The finding of the descending trend of CVD mortality is in agreement with some other reports around the world. For instance, in the study by Khan et al., it was revealed that the global mortality trend of ischemic heart disease has decreased slowly but progressively from 1990 to 2017 [24]. In another study, it was shown that the age-standardized mortality rate of stroke has decreased sharply by 33.4% over the same time period [25]. However, a study in Central Asia (which includes low- and middle-income countries) indicates that the trend of CVD mortality has increased during the past two decades. The authors concluded that this increment might be the result of insufficient preventive care, lack of awareness about the disease signs and symptoms, decreased physical activity, raised blood pressure, and underutilization of health care services [28]. Moreover, the results from a study by Movsisyan et al. suggest that the Central and Eastern European countries had the highest CVD mortality in Europe [29]. Overall, advances in treatment, improving the level of care, and controlling the risk factors of death in CVD patients (such as smoking, hypertension, and overweight) are the most important reasons for the reduction in the mortality rate of this disease [30].

In the current study, the MIR was used as a surrogate indicator for five-year survival rate of CVD patients. According to our findings, the overall trend of CVD MIR has continuously decreased from 0.41 in 1990 to 0.30 in 2017 with a slight slope (mean annual reduction) of − 0.004. Hopefully, the 26.8% reduction in mean global MIR implies that the 5-year survival of CVD patients had a noticeable rise over this 28-year period. However, looking at the MIR trend in the IHME super regions reveals that HI countries had the steepest fall (from 0.23 to 0.11 with more than 50% reduction) during these years, while countries in SA super region experienced the lowest reduction (from 0.44 to 0.40 with only 9% reduction) at the same time interval. It seems the wealthiest super region has promoted the survival rate of the patients much more quickly compared to the aforementioned regions. The key reasons for this rise in the survival rate are probably broader screening programs and the detection of disease at an earlier stage, promoting knowledge, attitudes, and practices of people around the world about strategies for preventing cardiovascular disease, and promoting improved level of care in CVD patients.

In the present research, the trends of CVD incidence, mortality, and MIR were compared between developed and developing countries for the period 1990-2017 via a marginal model which provides a more accurate evaluation of the longitudinal association between CVD burden and the development status of the region. Our findings demonstrate that the incidence rate reduction in developed countries is appreciably faster than that in developing regions (a slope ratio of 1.8) in the time period. Likewise, based on the reported descriptive statistics, CVD incidence in developed countries decreased by about 14.4% in this 28-year period, while the reduction in developing countries was only about 2.7% during the same time period. According to Feigin et al., the age-standardized incidence of stroke was diminished by 12% in developed countries, whereas it grew by 12% in developing areas between 1990 and 2010 [31]. The findings from the GBD study 2016 indicate that China and El Salvador had the highest and lowest age-standardized incidence of stroke in the period 1990–2016, respectively [27]. Also, the highest CHD incidence rates were mainly found in poor African countries such as Somalia and Burundi, while the lowest rates were mostly observed in developed countries such as Qatar, Portugal, and France [26]. A number of studies suggest that there are multiple factors behind the global reduction in CVD incidence. These factors could be adopting a healthier lifestyle, promoting the level of knowledge about CVD risk factors (like obesity, smoking, hypertension, and bad eating habits), considerable shift in socioeconomic status with increased urbanization and the adoption of western calorie especially in developing countries [32–34]. Nevertheless, in several countries, an increasing trend in CVD incidence has occurred. Namely, the incidence of CVD among South Korean patients continued to increase during the period 2013–2015 which might be explained by better access to diagnosis techniques, registration, and reports [35].

In this paper, it was found that both developed and developing countries experienced a fairly similar declining trend in CVD mortality from 1990 to 2017. Furthermore, based on descriptive statistics, it was observed that about 23.8 and 18.5% reduction in mortality rates have occurred in developed and developing countries, respectively. The most plausible explanations for the greater fall in more developed countries may be better control of risk factors, such as hypertension, obesity, hypercholesterolemia, and diabetes, promoting better care, using more effective treatments, and allocating higher budgets for health services in these areas [16, 36–38]. On the other hand, rapid economic transition, high productivity in the economy, and rising per capita income in several developing countries seems to play a crucial role in the reduction of CVD mortality rate over the past few decades [39, 40]. These findings are in agreement with some other research in this field. In a study by Kollia et al., they reported an approximate 43% decrease in CVD death rate in developed countries versus only 13% in developing nations between 1990 and 2013 [37]. Nevertheless, some conflicting results have been previously reported in different parts of the world. For example, a study in India showed a rapid increase in CVD mortality between 1990 and 2016. The authors have stated that this increase seems to be almost entirely owing to population aging in this Asian country. Nevertheless, high blood pressure, high total cholesterol, and high fasting blood glucose level were the major contributors to CVD mortality in this country [41].

Another noticeable finding is that even though the developed and developing countries underwent a statistically significant decline, the developing counties had a more rather shallow reduction in CVD MIR in comparison to developed ones during a 28-year follow-up. Nonetheless, the downward trend implies that the five-year survival rate of CVD patients was increased for both sets of countries. The significant relationship between HDI and MIR highlights that the countries focused on improving the adoption of a healthy lifestyle as well as access to health care services in either prevention or treatment of CVD, increasing life expectancy, and increasing urbanization during recent years. Socioeconomic status is directly correlated with a patient’s survival so that patients with higher socioeconomic conditions may experience greater survival [42]. Finally, it is necessary to highlight that the awareness of CVD has increased in several countries. This helps to improve early detection of signs and symptoms of CVD, better health care, and thereby superior prognosis.

### Strengths and limitations

In addition to the limitations reported in GBD studies, the present research encountered some potential limitations. Firstly, lack of precise and reliable data for incidence and mortality rates in some countries, especially in less developed regions, entails GBD to report the estimated rates. The second limitation is the small number of countries in some super regions which makes the statistical inference (for example, valid estimation of model parameters) challenging for them. In the previous decades, a number of studies have shown that occupational exposure to organic solvents, pesticides, silica dust, engine exhaust, welding fumes, arsenic, benzopyrenes, lead, dynamite, carbon disulfide, carbon monoxide, metalworking fluids, inorganic mercury, phenoxy acids, and electrolytic production of aluminum is significantly associated with different types of cardiovascular diseases [43–47]. In the present epidemiologic study, we did not aim to investigate the effect of occupational exposures on global burden of CVD. We believe that assessment of this association in all world countries needs more specialized researches by the experts in the future. This could be considered as one of the most important limitations of our study. In spite of the aforementioned limitations, our research is a novel and comprehensive study in this field. The main strength of the present study is the longitudinal nature of the GBD dataset with a reasonably long period of follow-up with 28 repeated measurements which enables us to obtain more accurate estimates. Considering this issue, we utilized a more complex and convenient statistical approach to reach more reliable inferences. This modeling approach gives us the opportunity to capture the longitudinal nature of response data and explanatory variables. However, previous studies have employed either univariate correlation coefficients or simple linear regression models to investigate the relationship between the burden of disease indices and other potential indicators like socio-economic measures. Lastly, using the MIR index as a proxy for survival appears to be a reliable approach to explain the discrepancies in age standardized rate of incidence and mortality from CVD among different geographic areas. To calculate this index, there is no need to conduct studies with long-term follow-up which are potentially time-consuming, expensive, and prone to different biases. Additionally, it should be noted that based on our literature review, there was no related research in this field (the relationship between development factor and CVD MIR). Hence, more comprehensive researches should be conducted to assess the cross-sectional and longitudinal association between the development factor and CVD MIR (or survival rate of the patients) more properly.

## Conclusions

In summary, we comprehensively described the global patterns of CVD incidence, mortality, and MIR and analyzed their temporal trends from 1990 to 2017 via an advanced statistical technique. Totally, it was found that the overall CVD incidence and mortality have significantly declined over the 28-year study period, while the mortality-to-incidence ratio was rather stable. This is an encouraging message for health policymakers among the total world population; since it reveals that global efforts for controlling the CVD burden (reducing the CVD incidence and mortality rates) were reasonably successful. In addition, the quite parallel lines for incidence, mortality, and MIR trends in developed and developing countries imply that the gap between these indices in the year 1990 remains fixed until the ending year of the study. In this context, both countries should put more effort into lowering these gaps while they attempt to decrease the slope of the CVD burden indices more rapidly in the upcoming years.

## Data Availability

We confirm that all methods were performed in accordance with the relevant guidelines and regulations. The datasets analysed during the current study are available in http://ghdx.healthdata.org/gbd-results-tool and http://hdr.undp.org/en/data.

## Abbreviations

CVD: Cardiovascular disease
WHO: World health organization
MIR: Mortality-to-incidence ratio
HDI: Human development index
GNI: Gross national income
GDP: Gross national product
IHME: Institute for health metrics and evaluation
GBD: Global burden of disease
YLL: Years of life lost
YLD: Years lived with disability
DALY: Disability adjusted life years
CEEECA: Central Europe, Eastern Europe, and Central Asia
HI: High Income
LAC: Latin America and Caribbean
NAME: North Africa and Middle East
SA: South Asia
SAEAO: Southeast Asia, East Asia, and Oceania
SSA: Sub-Saharan Africa
UNDP: United Nations Development Programme
GEE: Generalized estimating equations
CHD: Congenital heart disease
